# What is it like to be a choanoflagellate? Sensation, processing and behavior in the closest unicellular relatives of animals

**DOI:** 10.1007/s10071-023-01776-z

**Published:** 2023-04-17

**Authors:** Núria Ros-Rocher, Thibaut Brunet

**Affiliations:** Evolutionary Cell Biology and Evolution of Morphogenesis Unit, Institut Pasteur, Université Paris-Cité, CNRS UMR3691, 25-28 Rue du Docteur Roux, 75015 Paris, France

**Keywords:** Origin of animals, Choanoflagellates, Sensation, Multicellularity, Basal cognition

## Abstract

All animals evolved from a single lineage of unicellular precursors more than 600 million years ago. Thus, the biological and genetic foundations for animal sensation, cognition and behavior must necessarily have arisen by modifications of pre-existing features in their unicellular ancestors. Given that the single-celled ancestors of the animal kingdom are extinct, the only way to reconstruct how these features evolved is by comparing the biology and genomic content of extant animals to their closest living relatives. Here, we reconstruct the *Umwelt* (the subjective, perceptive world) inhabited by choanoflagellates, a group of unicellular (or facultatively multicellular) aquatic microeukaryotes that are the closest living relatives of animals. Although behavioral research on choanoflagellates remains patchy, existing evidence shows that they are capable of chemosensation, photosensation and mechanosensation. These processes often involve specialized sensorimotor cellular appendages (cilia, microvilli, and/or filopodia) that resemble those that underlie perception in most animal sensory cells. Furthermore, comparative genomics predicts an extensive “sensory molecular toolkit” in choanoflagellates, which both provides a potential basis for known behaviors and suggests the existence of a largely undescribed behavioral complexity that presents exciting avenues for future research. Finally, we discuss how facultative multicellularity in choanoflagellates might help us understand how evolution displaced the locus of decision-making from a single cell to a collective, and how a new space of behavioral complexity might have become accessible in the process.

**Supplementary Information:**

The online version contains supplementary material available at 10.1007/s10071-023-01776-z.

## Introduction

We are animals, and animal biology and behavior thus tend to capture a disproportionate share of the attention of biologists and of the general public alike. Yet, it is not only phylogenetic chauvinism that makes us think that animals are special. Defined as heterotrophic, obligately multicellular eukaryotes capable of phagocytosis and of global body deformations by collective cell contractility (Nielsen 2012), animals (or Metazoa) appear to be a unique evolutionary experiment. Indeed, although other complex multicellular eukaryotes have converged at least four times independently onto the algal lifestyle (red, green, brown and golden algae) and have also independently evolved twice into fungus-like forms (oomycetes and fungi proper), animal-like morphology and lifestyle evolved only once (Knoll 2011; Cavalier-Smith 2017). When it comes to behavior and cognition, the abilities of animals seem similarly unique. A clear and well-characterized example of animal-specific behavioral trait (in the current state of knowledge) is associative learning (or classical conditioning), which has been convincingly documented in multiple bilaterian animals (reviewed in (Loy et al. 2021) and some cnidarians (Cheng 2021). On the other hand, the many experimental attempts to discover it in non-metazoans such as plants or ciliates have either given negative results or tentative positive claims that do not seem to have withstood scrutiny or attempts at replication (see Gershman et al. (2021), Dussutour (2021) and references therein; but see Carrasco-Pujante et al. (2021) for a possible documentation of associative conditioning in amoebozoans).

**Fig. 1:**
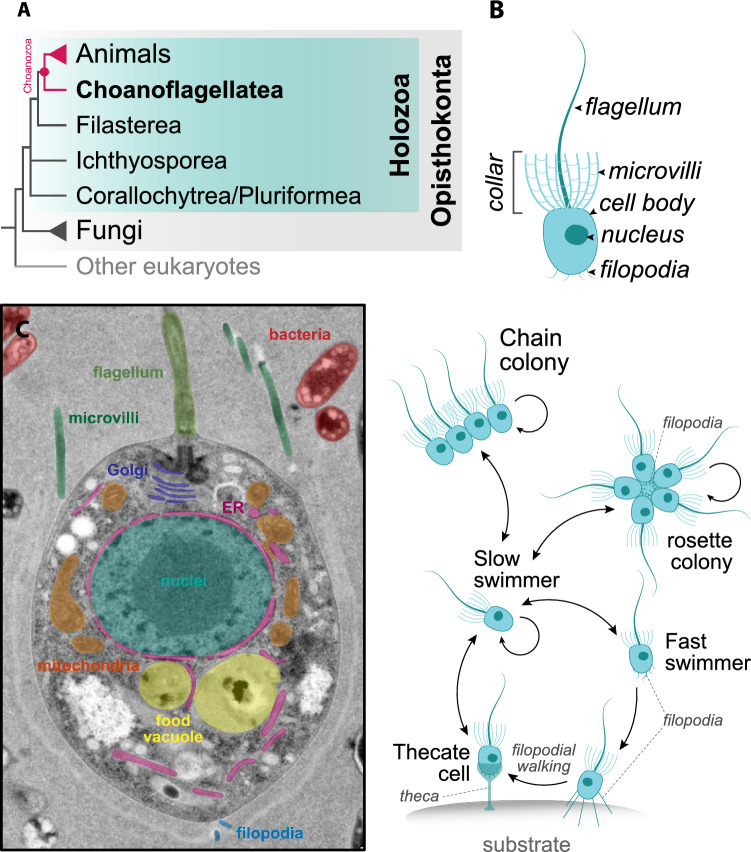
Phylogenetic position and cellular features of choanoflagellates. **A** Choanoflagellates (Choanoflagellatea) are the sister group to animals (Metazoa), forming a monophyletic clade named Choanozoa (pink). Animals, choanoflagellates and other unicellular relatives form the Holozoa clade (blue) within the Opisthokonta eukaryotic supergroup (gray). Topology based on King et al. (2008), Fairclough et al. (2013), Torruella et al. (2015), Hehenberger et al. (2017), and Grau-Bové et al. (2017). Uncertain relationships are depicted as polytomies. **B** Diagnostic features of a choanoflagellate cell. **C** Transmission electron micrograph of a *Salpingoeca rosetta* cell showcasing the generic cellular architecture of a choanoflagellate. Colored labels refer to the nearby cellular structure of the same color, including the apical flagella and collar (which together form the collar complex), organelles, and basal filopodia. ER: endoplasmic reticulum. Image modified from Booth et al. (2018). **D** Life stages of the colonial *S. rosetta* (Dayel et al. 2011; Levin and King 2013). The single-celled slow-swimmer stage can transition to fast swimmers and to clonal multicellular forms (chain and rosette colonies), where neighboring cells are linked by intercellular bridges. Fast swimmers can also transition to a sessile thecate stage through a process named “filopodial walking”. In this process, basal filopodia contact the substrate and allow the cell to adhere, move and settle at a given spot, where it then secretes an organic covering known as theca. Arrows indicate directionality of each transition (loop arrows indicate cell division)

If one considers the phylogenetic tree of eukaryotic life, however, animals occupy but a small, inconspicuous twig (Burki et al. 2020). Animals are evolutionarily related to a disparate set of microbes – mostly flagellates, amoebae or spherical cells – that resemble, at first sight, many other protists in form and lifestyle (Fig. 1A). The immediate sister group of animals are the choanoflagellates (King and Carroll 2001; King et al. 2008; Ruiz-Trillo et al. 2008; Carr et al. 2008; Grau-Bové et al. 2017), a group of ovoid bacterivorous flagellates that are globally distributed in aquatic environments (Brunet and King 2017). Their signature feature is a “collar complex” – a ring of fine and rigid appendages called “microvilli” that surround their beating flagellum (Fig. 1B, C) (Leadbeater 2014). These two types of appendages cooperate to allow choanoflagellates to capture the bacteria they feed on: the beating of the flagellum creates a flow that carry bacterial prey in the liquid, and the microvilli act as a filter that first traps and then transports them toward the cell body to be phagocytosed. The next closest unicellular relatives of animals are the filastereans (a group of filopodiated amoebae and predatory amoeboflagellates), followed by the ichthyosporeans (amoeboid, flagellated or spherical cells that develop from large multinucleated cells), and the corallochytreans/pluriformeans (fungus-like osmotrophs or predatory amoeboflagellates) (Sebé-Pedrós et al. 2017; Ros-Rocher et al. 2021). The ancestry of animal behavioral traits, including the mechanisms for sensation and cognition, can only be reconstructed by comparing the biology and genome content of animals with those present in these close relatives.

Here, we summarize what is currently known (and, perhaps more importantly, what remains to be discovered) about the perceptive universe of choanoflagellates – their *Umwelt* (Uexküll 1934; Yong 2022). Although choanoflagellates can readily be isolated from most aquatic environments, relatively little is known about their behavior in the wild. As for most other microbes, their minute size and the highly granular and ephemeral nature of aquatic microenvironments (Stocker et al. 2008; Stocker 2012) conspire to make in situ observation challenging. However, laboratory studies based on simplified reconstituted environments (notably using microfluidics (Kirkegaard et al. 2016a; Miño et al. 2017)) have revealed part of the sensory and behavioral repertoire of choanoflagellates, and have demonstrated the presence of chemosensation, photosensation and mechanosensation. An independent source of evidence has been comparative genomics, which has revealed putative molecular chemoreceptors, photoreceptors and mechanoreceptors in the genomes of choanoflagellates – many (but not all; see ﻿Sect. “﻿Photosensation”﻿) of which are homologous to animal sensory receptors. This rich molecular repertoire provides a potential basis for the few well-described behaviors, but also suggests that those might be but the tip of the iceberg. Taken together, behavioral and genomic evidence in choanoflagellates thus support the existence of unicellular precursors to the animal senses of taste/smell, touch and sight.

## Detecting stimuli: senses and sensory receptors in choanoflagellates

The response to a stimulus by a cell or organism involves a series of consecutive events: (i) the detection of the stimulus by a molecular receptor; (ii) the conversion or transduction of the stimulus into biochemical activity of a cellular signaling pathway; and (iii) a functional output or *behavior* (see Sect. “Responding to stimuli: processing and behavioral responses in choanoflagellates”).

Choanoflagellates navigate complex aquatic environments which expose them to variable distributions of potential stimuli, including mates, food (bacteria), predators and abiotic parameters (e.g., oxygen levels, pH, light and temperature). To these varied external stimuli corresponds an equal diversity of behavioral outputs (such as directed locomotion in several classes of environments, settlement, feeding, swarming and mating). The conversion of stimuli to responses in choanoflagellates is incompletely understood but is already known to involve chemosensory, photosensory and mechanosensory pathways, each of which likely involves distinct molecular machineries that will be reviewed below.

### Chemosensation

#### Chemical stimuli detected by choanoflagellates

Chemosensation is the perception of environmental chemicals, including both abiotic and biotic molecules. Aquatic environments such as those inhabited by choanoflagellates can vary considerably in their physico-chemical parameters (e.g., dissolved oxygen concentration, pH and temperature) and availability of food source (bacteria) even at short spatial scales (Stocker 2012). Choanoflagellates display abundant mitochondria (Fig. 1C) (Leadbeater 2014; Laundon et al. 2019), which suggests they rely on aerobic metabolism to produce ATP and perform cellular functions. Thus, oxygen content as well as chemical cues indicating the presence of bacteria are likely relevant to their existence and logical prime candidates for chemosensation.

#### Oxygen

**Fig. 2:**
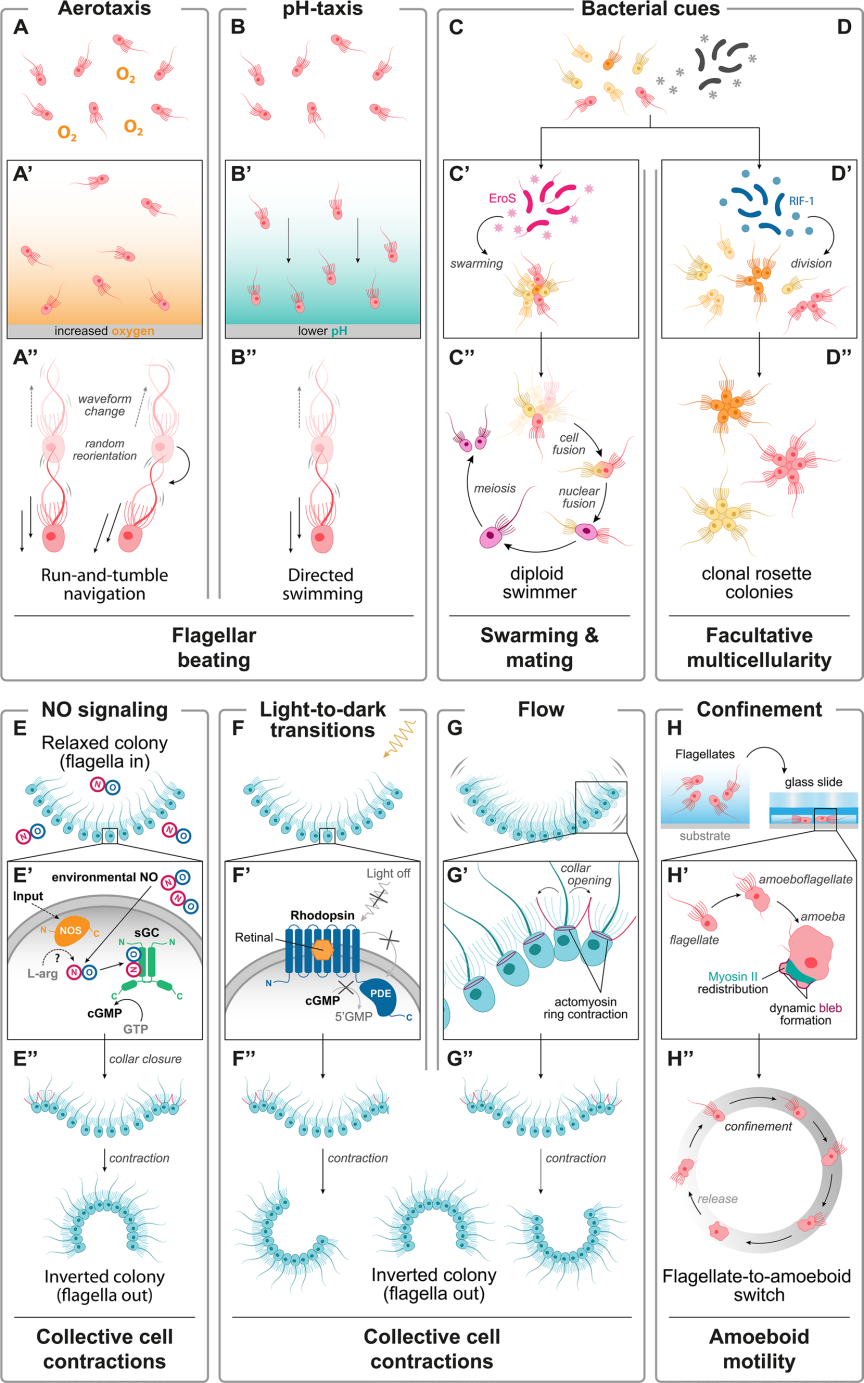
External stimuli and choanoflagellate cellular responses by chemosensation, photosensation and mechanosensation. (**A**–**H**) Choanoflagellate responses to various external stimuli, involving chemosensation (**A**, aerotaxis (Kirkegaard et al. 2016a); **B**, pH-taxis (Miño et al. 2017); **C**–**D**, bacterial cues (Alegado et al. 2012; Woznica et al. 2016, 2017); **E**, NO signaling ((Reyes-Rivera et al. 2022)), photosensation (**F**, light-to-dark transitions (Brunet et al. 2019)) and mechanosensation (**G**, flow (Leadbeater 1983a); **H**, confinement (Brunet et al. 2021)). **A’**–**H’** Conversion of stimuli to cellular responses, including, when known, sensory receptors. **A’’**–**H’’** Behavioral outputs resulting from chemosensation, photosensation and mechanosensation

Two recent studies relying on microfluidics to create spatially and temporally controlled microenvironments have shown that choanoflagellates can detect both dissolved oxygen and bacteria. The first study showed that the choanoflagellate *Salpingoeca rosetta* displays positive aerotaxis (i.e., migration towards higher oxygen concentrations) in both multicellular (rosette colony) and unicellular (fast swimmer) forms (Figs. 1D, 2A) (see Sect.  “Flagellar beating”) (Kirkegaard et al. 2016a). *S. rosetta* seems to be able to sense and respond to oxygen gradients across at least 3 orders of magnitude, which suggests that *S. rosetta* is more sensitive to small changes in oxygen levels at low oxygen concentrations, and therefore, that its sensitivity to oxygen might be logarithmic rather than linear. Interestingly, logarithmic sensing of diverse stimuli is a common strategy in many animal senses (where the principle of logarithmic response is sometimes referred to as “Weber’s law” (Jeans 1968)) and also underlies chemotaxis in the bacterium *Escherichia coli* (which is perhaps the organism in which the molecular mechanism of chemotaxis has been dissected in the most detail (Block et al. 1983)).

#### pH

Microfluidic assays have also shown that *S. rosetta* displays clear chemotaxis toward a band containing the bacterial prey *Algoriphagus machipongonensis* (Miño et al. 2017). Surprisingly, the attracting molecule did not turn out to be a bacterium-specific cue, but rather acidification of the medium caused by bacterial metabolism, which resulted in a significant drop in pH (from 8 to 6–7) (Fig. 2B). In the absence of a difference in pH, *S. rosetta* entirely lost the ability to detect bacteria. Interestingly, pH-taxis seems to rely on directed swimming (see Sect.  “Flagellar beating”) and is only exhibited by fast swimmers (the dispersal form of choanoflagellates), but not by rosette colonies.

#### Nitric oxide

**Fig. 3:**
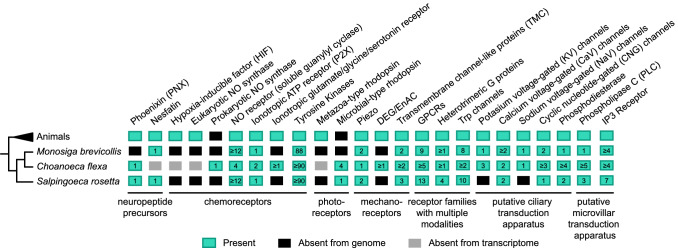
Phylogenetic distribution of putative molecular sensors and sensory transducers in choanozoans. Presence or absence of key chemosensory, photosensory and mechanosensory receptors as well as components of signal transduction pathways are represented in columns and color-coded (see key in figure). Numbers inside each box represent number of ortholog sequences either previously reported in literature or predicted by ourselves. Note that experimental evidence of these molecules in the sensory categories listed requires further investigation. See Table S1 for methods of ortholog identification and accession numbers. The phylogenetic relationships of selected taxa are based on Brunet et al. (2019)

Another small inorganic molecule choanoflagellates can sense is the diatomic gas nitric oxide (NO), which acts as a hormone in virtually all extant animal lineages and likely did so in the Urmetazoan (Colasanti et al. 2010; Andreakis et al. 2011; Moroz et al. 2020). Treatment of colonies of the multicellular choanoflagellate species *Choanoeca flexa* with exogenous NO causes global inversion of the colony that is thought to represent an escape response (Fig. 2E) (Reyes-Rivera et al. 2022). The source of NO in natural conditions is unclear: NO can be produced by bacterial metabolism in the ocean (Cutruzzolà 1999; Martens-Habbena et al. 2015) but might also be generated by choanoflagellates themselves, since an NO synthase has been identified in the transcriptome of *C. flexa* (alongside an NO receptor) (Fig. 3). This raises the possibility that NO signaling might mediate intercellular communication in this species, either within or between colonies (Reyes-Rivera et al. 2022)*.*

#### Organic molecules serving as specific molecular signatures of other species

Besides these small inorganic molecules, choanoflagellates are also able to detect complex organic molecules that serve as signatures of specific species in their biotic environment. These species include potential mates, competitors, preys, predators, pathogens, and symbionts. Although the identity of these ecological partners has long been elusive, some have recently been discovered. For example, the bacterium *Vibrio fischeri* induces swarming and mating in *S. rosetta* through the activity of the secreted chrondroitinase EroS (Extracellular Regulator of Sex) (Fig. 2C) (Woznica et al. 2017). Specificity of response to bacterial prey is also evident in the control of multicellular development in *S. rosetta*, which is induced by sulfonolipids (named Rosette-Inducing Factors, or ‘RIFs’) secreted by the bacterium *A. machipongonensis* (Fig. 2D) (Alegado et al. 2012; Woznica et al. 2016). Choanoflagellate predators include ciliates, amoebae, and heliozoans (Kumler et al. 2020; Koehl 2021); it remains to be discovered whether choanoflagellates respond to cues secreted by these predators. Recently, the bacterium *Pseudomonas aeruginosa* has been identified as a pathogen of the choanoflagellate *Monosiga brevicollis*. *M. brevicollis* selectively avoids phagocytosing *P. aeruginosa*, which suggests that choanoflagellates might discriminate between distinct bacterial species, presumably by sensing surface chemicals (Woznica et al. 2021). Finally, genomic data indicate that choanoflagellates can be infected by giant viruses (Needham et al. 2019) and harbor symbiotic or closely associated bacteria (Hake et al. 2021; Needham et al. 2022). It is unclear whether these interactions mediate chemosensation, which presents an avenue for future research.

#### Chemical cues from conspecific choanoflagellates

The biotic environment of choanoflagellates also includes other individuals of the same species, and conspecific choanoflagellates might thus exchange signals. A plausible biological context is mating, which occurs in choanoflagellates under the form of cell–cell fusion (Levin and King 2013). This process might thus involve guidance toward, and recognition of, sexual partners by chemical cues such as pheromones. As previously mentioned, the bacterium *V. fischeri* induces swarming and mating in *S. rosetta* through the activity of EroS, in a way that suggests it might mimic the activity of a (hypothetical) inducer of mating secreted by the choanoflagellates themselves (Woznica et al. 2017). Other candidate chemicals for intraspecific, intercellular communication between choanoflagellate cells have recently been identified in the genome of *S. rosetta:* two neuropeptide precursors (phoenixin (PNX) and nesfatin) belonging to a class of secreted intercellular signals that are deeply conserved across animals and classically act through G-protein-coupled receptors (GPCRs, Fig. 3) (Yañez-Guerra et al. 2022). The effect of these neuropeptide precursors on choanoflagellate behavior remains unknown but will surely be the subject of future research. Although little is otherwise known about secretion or signal detection in choanoflagellates, their genomes encode homologs of many animal pre-synaptic and post-synaptic proteins and cell biological evidence indicates the likely presence of a polarized secretion apparatus below the apical pole (Burkhardt et al. 2011, 2014; Burkhardt 2015; Colgren and Burkhardt 2022). Moreover, many homologs of animal sensory receptors have been detected in choanoflagellate genomes and transcriptomes (see below).

#### Chemoreceptors and transduction pathways in choanoflagellates

The receptors and signaling pathways underlying the aforementioned choanoflagellate chemosensory responses remain incompletely known and will likely be the subject of future research. The mechanism of oxygen sensing, for example, remains elusive: in animals, oxygen is sensed by the Hypoxia-inducible factor (HIF) transcription factor, which does not exist in choanoflagellates (and would likely act too slowly to modulate swimming during aerotaxis) (Fig. 3) (Mills et al. 2018).

Some insights might be gained, however, by comparing known animal chemosensors to those predicted to be encoded by the genomes and transcriptomes of choanoflagellates. In animals, environmental chemicals are often detected by two classes of receptors: (1) G which for example mediate olfaction by ciliated sensory neurons in mammals (Jenkins et al. 2009) and in the nematode *Caenorhabditis elegans* (Bargmann 2006; Vidal et al. 2018); and (2) receptor-ion channels of the Transient receptor potential channels (Trp) family, which tend to broadly detect nociceptive stimuli including chemical ones (such as acidity) but also some mechanical or thermal stimuli. Both families are of ancient eukaryotic ancestry and abundantly represented in choanoflagellates (Fig. 3) (de Mendoza et al. 2014; Peng et al. 2015). For instance, the genome of *S. rosetta* encodes 10 predicted Trp channels belonging to 5 families shared with metazoans (TrpA, TrpC, TrpM, TrpML and TrpV) (Cai 2008; Peng et al. 2015; Himmel and Cox 2020) as well as a dozen predicted GPCRs (de Mendoza et al. 2014).

Animal cells respond not only to environmental chemicals but also to molecules secreted by other cells within the organism, such as hormones and neurotransmitters. Intriguingly, several bilaterian neurotransmitters are generic biomolecules that appear to have been co-opted for intercellular communication during the evolution of the nervous system (Arendt 2020; Moroz et al. 2021), including ATP (the universal energetic currency of cells), glutamate and glycine (amino acids), and serotonin (a tryptophan derivative present in most living cells, where it might generally act as an antioxidant (Azmitia 2020)). These four molecules can be detected by channel receptors which all have putative homologs in choanoflagellate genomes and/or transcriptomes, and thus evolved before the nervous system (Fig. 3 and references in the legend). Yet, biochemical characterizations will be necessary to test whether the choanoflagellate homologs of those animal receptors bind the same ligands, and in what biological context they act. A tantalizing possibility is that the presence of normally intracellular biomolecules in the environment might indicate the neighboring presence of damaged or dying cells, and thus serve as a “danger signal” (Moroz et al. 2021) – which remains to this day one of the functions of extracellular ATP in mammals (Trautmann 2009) and land plants (Tanaka et al. 2014).

Besides small hormones/neurotransmitters, animal cells can detect other intercellular signaling molecules via GPCRs (which thus do not only detect environmental chemicals) and receptor tyrosine kinases (Alberts 2008). A surprising finding from choanoflagellate genomes was that they harbored an extensive repertoire of tyrosine kinases that even exceeded that of some animals (King and Carroll 2001; Suga et al. 2008, 2012; Manning et al. 2008; Prieto-Echagüe et al. 2011; Miller 2012), including many receptor tyrosine kinases (Fig. 3) (King et al. 2008; Manning et al. 2008). Although none of the ligands of those receptors have yet been identified, that extensive molecular diversity might suggest a role in discriminating surrounding species in the biotic environment of choanoflagellates. Excitingly, functional characterization of the choanoflagellate complete set of protein kinases (a.k.a., the kinome) has recently been initiated (Rutaganira et al. 2022), and has already shown activation of p38 kinase in *S. rosetta* by environmental stressors (i.e., heat shock and oxidative stress).

Although functional characterization of the choanoflagellate chemosensory pathways is still in its infancy, the advent of functional genetics in choanoflagellates (Booth et al. 2018; Booth and King 2020; Woznica et al. 2021) might in the future allow de-orphanization (i.e., identification of the ligands) of those receptors and identification of the downstream functional outputs.

### Photosensation

Almost all primary production of biomass on earth relies on photosynthesis. For this reason, light represents an important cue both for photoautotrophs (which harness it as a source of energy) and for heterotrophs (which feed on biomass directly or indirectly produced by photosynthesis). Indeed, photosensation (i.e., the process of sensing light) is near-universal across living organisms, including all animal lineages (Nilsson 2009).

In choanoflagellates, response to light had long been predicted based on comparative genomics: the genome of *S. rosetta* encodes a candidate photoreceptor, a microbial-type rhodopsin fused to a phosphodiesterase (Fig. 3) (Yoshida et al. 2017; Tian et al. 2018). In vitro biochemical assays have shown that the enzymatic activity of the phosphodiesterase moiety is activated by light. Microbial-type rhodopsins homologous to the one of choanoflagellates are broadly found in bacteria and eukaryotes other than animals, in which they perform diverse functions: for example, light-driven ion pumps, light-gated ion channels, positive and negative phototaxis sensors, and light-activated enzymes (Jung et al. 2003; Ernst et al. 2014; Brown 2014; Grote et al. 2014; Inoue et al. 2015). They notably mediate photosensation in green algae and in unicellular fungi, suggesting that they might already have done so in the last eukaryotic common ancestor (Avelar et al. 2014; Brunet et al. 2019; Galindo et al. 2022). The fusion between microbial rhodopsins and phosphodiesterases, on the other hand, seems unique to choanoflagellates and is conserved across much of choanoflagellate diversity (Brunet et al. 2019). Perplexingly, microbial rhodopsins have been lost in animals, which detect light instead with metazoan-type rhodopsins (specialized GPCRs without clear homology to microbial rhodopsins (Fig. 3) (Shichida and Morizumi 2006; Palczewski 2006; Hofmann et al. 2009; Mackin et al. 2014; Koyanagi and Terakita 2014)). Thus, the ancestral photosensation system seems to have been replaced by a different one during early animal evolution. The ecological significance of this replacement, if any, is still unclear.

Recently, the first in vivo evidence for photosensation in choanoflagellates came with the discovery of *C. flexa*, whose large contractile colonies display a reflex that inverts their global curvature in response to sudden darkness (Fig. 2F). Biochemical evidence suggests that the molecular photosensor controlling inversion is a choanoflagellate-specific rhodopsin-phosphodiesterase, of which the *C. flexa* transcriptome encodes four (Brunet et al. 2019). The ecological function of this response is unclear, but inversion appears to mediate a trade-off between feeding and swimming behavior. Moreover, light-controlled inversion has been shown to allow photokinesis, i.e., accumulation in lit areas due to faster swimming in darkness and cessation of motility under light (Brunet et al. 2019; Jékely 2019). The molecular mechanisms through which phototransduction activates contractility remains to be discovered, but efforts to develop genetic tools in this species might make functional studies possible in the near future.

### Mechanosensation

Mechanosensation is the transduction of mechanical stimuli detected by molecular mechanoreceptors into intracellular signaling pathways. This sensory mechanism is present in all animal lineages, including sponges, cnidarians, placozoans and bilaterians (Fritzsch et al. 2007; Fritzsch and Straka 2014; Mah and Leys 2017). In humans, it provides the basis for the senses of hearing and touch, among others (such as vestibular and kinesthetic senses). The first observers of choanoflagellates already noticed that they were sensitive to mechanical stimuli, and that flow induced a rapid contraction of the collar (James-Clark 1867; Saville-Kent 1880). A similar response can be induced by gentle touch of the collar of *Choanoeca perplexa* with a micromanipulated needle or by flow from a microinjector (Leadbeater 1983b). In the multicellular form of *C. flexa*, mechanical stimulation by shaking of culture flasks also induces rapid collar contractions and overall colony inversion (Fig. 2G), just like light-to-dark transitions (Reyes-Rivera et al. 2022).

Recently, a more intense type of mechanical stimulus has been tested in choanoflagellates: whole-cell confinement (i.e., entrapment of cells in a space narrow enough to deform the cell body)*.* Confined *S. rosetta* cells undergo a dramatic phenotypic remodeling within seconds, in which they retract their flagellum, scatter (and eventually resorb) their microvilli, and extend cellular protrusions that allow them to crawl out of the confined space in a steady and directional fashion, and regain liquid environments, after which their collar is regenerated (Fig. 2H). This is referred to as a “flagellate-to-amoeboid switch” (Brunet et al. 2021). This switch seems conserved across choanoflagellate diversity (Brunet et al. 2021) and may allow escape from natural confined microenvironments (e.g., silts, which are fine-grained interstitial media) or even capture by predatory microeukaryotes.

The molecular sensors involved in the diverse choanoflagellate mechanoresponses have not yet been identified. In animals, sensory mechanoreceptors are diverse and include several receptor families (Fig. 3). For instance, touch in vertebrates seem to rely near-entirely on the recently discovered Piezo channels (Ranade et al. 2014; Walsh et al. 2015; Murthy et al. 2017; He et al. 2018). Vertebrate hearing, on the other hand, relies on a different family of mechanosensory channels named Transmembrane channel-like (TMC) (reviewed in (Al-Sheikh and Kang 2020)). In nematodes, sensory mechanoreception relies on Degenerins and the epithelial amiloride-sensitive Sodium channel (DEG/ENaC) or TrpC channels, depending on the cell type (Geffeney and Goodman 2012). Molecular mechanosensors are less well identified in other species and cell types.

Interestingly, these families of metazoan mechanosensory channels all have homologs in choanoflagellates including five subfamilies of Trp channels, TMC, Piezo, and DEG/ENaC (Fig. 3). Although none of these families has yet been functionally characterized in choanoflagellates, this molecular diversity reinforces the behavioral evidence for the existence of several types of sensory mechanotransduction in choanoflagellates, and suggests that some mechanosensitive behaviors and cellular structures might remain to be discovered.

## Responding to stimuli: processing and behavioral responses in choanoflagellates

Following transduction and perception of the stimuli, the final step is the generation of a cellular response. For the purpose of this review, we will define *behavior* as any active, controlled, and reversible change in the shape, posture or activity of organisms within their environment. In choanoflagellates, most behaviors are implemented by a small set of core cellular effectors, including flagellar beating, local or global actomyosin contractility, filopodial extension/retraction, and remodeling of the extracellular matrix to modulate adhesion. Interestingly, all of these effectors are also known in some animal cell types, suggesting they might have been present in the last choanozoan common ancestor.

### Flagellar beating

Flagella (or cilia) are ancient sensorimotor organelles which mediate both sensation and locomotion in a broad range of eukaryotes and likely did so in the last eukaryotic common ancestor (Mitchell 2007). Directed locomotion is often achieved via a change of flagellar beating, from symmetric (straight swimming) to asymmetric (turning) (Fig. 2A’’, B’’) (Inaba 2015). Choanoflagellate flagellar beating remains little studied functionally, but its modulation is likely to provide the basis for chemotaxis (Fig. 2A, B). Interestingly, mathematical analysis of cellular trajectories under imposed gradients of stimuli suggests that aerotaxis and pH-taxis in *S. rosetta* might rely on different mechanisms. Specifically, aerotaxis in *S. rosetta* colonies relies on stochastic “run-and-tumble” navigation – i.e., alternation between phases of straight swimming and stochastic reorientations (a.k.a., kinesis) – rather than on directed swimming towards oxygen-rich regions (*taxis* proper) (Kirkegaard et al. 2016a). Consistently, *S. rosetta* cells seem to modulate direction rather than speed of swimming during this process, which might involve distinct flagellar waveforms (Fig. 2A). Thus, “﻿aerotaxis”﻿ in *S. rosetta* seems to be a form of kinesis, while pH-taxis apparently relies on directed swimming (similar to sea urchin sperm) and is thus taxis proper (Fig. 2B) (Jikeli et al. 2015; Miño et al. 2017). This suggests active modulation of flagellar beating in choanoflagellates. Moreover, flagellar beating might also differ between distinct *S. rosetta* cell types, as indicated by the fact that slow swimmers display circular or spiral trajectories, while fast swimmers swim in straight lines (Miño et al. 2017).

The signaling pathways that control flagellar beating in choanoflagellates are unknown, but some sensory molecules seem to localize to cilia in a conserved fashion across eukaryotes. In metazoans, sensory cilia are broadly found in chemosensory cells, photosensory cells and mechanosensory cells (Arendt 2003; Jenkins et al. 2009; Geffeney and Goodman 2012; Inglis et al. 2018; Bezares-Calderón et al. 2018). Animal ciliary sensation often relies on cyclic nucleotide signaling in downstream GPCRs: this is true in vertebrate and nematode chemosensory neurons (Bargmann 2006; Jenkins et al. 2009; Geffeney and Goodman 2012; Inglis et al. 2018), as well as in ciliary photoreceptors of the vertebrate retina (Arendt 2003). Intriguingly, comparative genomics suggests that the association between cilia/flagella and cyclic nucleotide signaling might be much more ancient than animals, as genes encoding components of cyclic nucleotide signaling correlate with the presence of cilia/flagella across the entire eukaryotic phylogenetic tree (Johnson and Leroux 2010). Little is known about cyclic nucleotide signaling in choanoflagellates (besides its involvement in *C. flexa* inversion (Brunet et al. 2019)), but the flagellar proteome of *S. rosetta* shows an enrichment for cyclic nucleotide phosphodiesterases (Fig. 3) (Sigg et al. 2017).

Another second messenger often involved in the modulation of flagellar beating is calcium, which controls ciliary reversal or arrest in model systems ranging from *Chlamydomonas* to ciliates, sperm cells, and animal multi-ciliated epithelia (reviewed in (Brunet and Arendt 2016)). In *Chlamydomonas*, the calcium-permeant mechanosensory channel Trp11 is located at the base of the flagellum and gives rise to calcium spikes in response to touch. Voltage-gated channels within the flagellar membrane then convert these calcium spikes into action potentials (i.e., rapid and brief depolarizations that propagate across the plasma membrane) (Harz and Hegemann 1991; Holland et al. 1997; Fujiu et al. 2009, 2011). Interestingly, voltage-gated channels also occur in the cilia of ciliates, and the phylogenetic distribution of voltage-gated channels seems to correlate with that of flagella/cilia in eukaryotes, which suggests that the control of flagellar beating by action potentials might extend beyond *Chlamydomonas* (Brunet and Arendt 2016).

In choanoflagellates, calcium signaling and electrophysiology still remain functionally uncharacterized. Some hints, however, come from comparative genomics: choanoflagellate genomes encode at least five families of Trp channels and predicted voltage-gated channels permeant to potassium (KV), calcium (CaV) and sodium (NaV) (Fig. 3) (Cai 2008; Liebeskind et al. 2011; Moran et al. 2015; Peng et al. 2015; Himmel and Cox 2020). Interestingly, calcium signaling and cyclic nucleotide signaling are not always two fully independent pathways; indeed, cyclic nucleotide-gated (CNG) channels can be permeant to calcium (Jikeli et al. 2015) and are present in choanoflagellates (Fig. 3) (Cai 2012). Functional analyses will be required to test the role of these molecules in the control of flagellar beating.

### Collar contractions

The collar complex is the signature feature of choanoflagellates. It is composed of an apical flagellum surrounded by a collar of interconnected microvilli. Microvilli mediate capture of bacteria and have also been co-opted for intercellular adhesion in the genus *Choanoeca* (Leadbeater 1983a; Brunet et al. 2019). There are indications that microvilli, like the flagellum, are both sensory and motile. As indicated above, the first observations of collar motility (in the form of collar contraction) in response to mechanical stimuli date back to the nineteenth century (James-Clark 1867; Saville-Kent 1880). These observations have been confirmed in the more recent literature (Leadbeater 1983b; Brunet et al. 2019). These contractions mediate retraction or avoidance in unicellular forms but also underlie multicellular shape changes in the genus *Choanoeca* (Fig. 2E”, F”, G”).

At the molecular level, collar contractions seem to rely on apical actin and myosin in choanoflagellates (Brunet et al. 2019) as cellular contractions generally do in animals (Levayer and Lecuit 2012). Interestingly, apical contractility is widespread in animal epithelial cells, both in embryos (Martin and Goldstein 2014) and in some adults, for example, the placozoan *Trichoplax adhaerens* (Armon et al. 2018). The pathways that control apical constrictions in choanoflagellates remain unknown but seem to involve cyclic guanosine monophosphate (cGMP) in *C. flexa*. Intriguingly, a recent study in *S. rosetta* demonstrated that biochemical inhibition of p38 kinase transiently induces an apical constriction-like behavior (Rutaganira et al. 2022). This suggests that kinase signaling might be involved in the control of choanoflagellate apical constriction, which remains to be tested in response to environmental stimuli.

In metazoans, microvilli, just like cilia, are often sensory and are notably involved in mechanosensation, chemosensation and photosensation. Mechanosensory microvilli underlie hearing in the hair cells of the mammalian inner ear (where they are confusingly named “stereocilia”) (Fritzsch et al. 2007; Fritzsch and Straka 2014). Mechanosensation in the hair cells of the inner ear relies on mechanosensory channels whose nature was long unclear but which seem to have recently been identified as TMC channels (Corey and Holt 2016; Pan et al. 2018; Jia et al. 2020; Al-Sheikh and Kang 2020). In many other metazoans, epidermal mechanosensory cells that detect the vibration of the medium display a collar complex similar to the one of choanoflagellates, with a central cilium surrounded by microvilli – suggesting a frequent involvement of both cilia and microvilli in mechanosensation across animal diversity (Brunet and King 2017; Bezares-Calderón et al. 2018, 2020).

Besides mechanosensation, microvilli mediate chemosensation in vertebrate taste buds (Höfer and Drenckhahn 1999; Smith and Margolskee 2001) as well as photosensation by rhabdomeric photoreceptors, which mediate vision in many invertebrates (Arendt 2003). Interestingly, microvillar chemosensation and photosensation both feed into the same pathway: GPCR/Gq signaling, which activates phospholipase C (PLC) and hydrolyzes the plasma membrane lipid PIP3 into IP3 and diacylglycerol. This in turn causes either calcium release (via IP3 acting on the reticulum, in chemosensory cells (Alberts 2008)) or membrane buckling detected by mechanosensory calcium channels (in fly photoreceptors (Hardie and Franze 2012)).

Notably, all the molecular components of the metazoan microvillar sensory transduction apparatus (TMC, GPCRs, heterotrimeric G proteins, PLC and IP3 receptor) are found in choanoflagellate genomes, suggesting a potential pre-metazoan origin of these sensory pathways (Fig. 3) (Cai and Clapham 2012; Fairclough et al. 2013; de Mendoza et al. 2014; Burkhardt et al. 2014; Erives and Fritzsch 2020; Rosa et al. 2021). Functional evidence, however, remains absent.

### Swarming

One of the most spectacular and unexpected behaviors reported in choanoflagellates in the past few years has been sexual swarming (Fig. 2C’’). As explained above, mating in the choanoflagellate *S. rosetta* was serendipitously found to be induced by the chondroitinase EroS secreted by the bacterium *V. fischeri* (Woznica et al. 2017). EroS triggers cellular aggregation into large swarms composed of dozens of cells that then undergo mating by each fusing with a partner (Fig. 2C) (Woznica et al. 2017). Moreover, bacterial chondroitinase can induce *S. rosetta* mating at environmentally plausible concentrations, suggesting that chondroitinase-producing bacteria could likely regulate choanoflagellate mating in nature.

The cellular mechanism of swarming represents a fascinating open question. Does it rely on chemotaxis – directed swimming of cells toward each other – in which case modulations of flagellar beating are likely involved? Or does it rely instead on (or in addition to) changes in adhesions keeping cells together – for example, downstream of controlled remodeling of the extracellular matrix? The latter is suggested by the occurrence of clumping (that at least at first sight resembles swarming) in *S. rosetta* glycosyltransferase mutants that show impaired organization of the extracellular matrix (Wetzel et al. 2018). Another possibility, finally, is that swarming is simply mediated by a failure to detach: the collar of choanoflagellates is generically “sticky” and readily captures bacteria and microbeads – suggesting that detachment following accidental contact between the collar of a choanoflagellate and the body of another might be an active process. Inhibition of detachment might thus be sufficient to promote swarming. Future research might help distinguish between those possibilities and may further clarify cell–cell recognition mechanisms guiding mating in choanoflagellates.

### Amoeboid motility

As stated above, choanoflagellates subjected to confinement transition from a flagellate to an amoeboid state (Fig. 2H). Amoeboid motility seems to rely on contractions of the actomyosin cytoskeleton that underlies the plasma membrane of the whole cell (known as the “cortex”) (Brunet et al. 2021). This global contractility differs from the local contractility of the collar discussed above, suggesting the coexistence of different – and independently controlled – cortical contractile systems within the cell.

An open question is whether choanoflagellate amoeboid motility is directed. Choanoflagellates presented with a boundary between confined and non-confined environments engage in directional crawling toward the shortest escape path, suggesting guidance by mechanosensation. Whether crawling can also be oriented by chemosensation remains untested. Finally, the molecular mechanosensors underlying the amoeboid switch itself remain to be discovered.

### Filopodial walking

Filopodia are slender actin-based cytoplasmic projections that allow sensing, cell–cell interactions and cellular migration (Mattila and Lappalainen 2008). In choanoflagellates, filopodia are located at the basal side of the cell in different cell stages, including swimming and thecate cells and rosette colonies (Sebé-Pedrós et al. 2013). Perhaps one of the least-known, but most intriguing, choanoflagellate behaviors is filopodial walking during the swimmer-to-thecate transition (Fig. 1D). Prior to settlement, choanoflagellates extend basal filopodia that contact environmental surfaces and engage in a short “walk” mediated by dynamic extension and retraction of filopodia (whose mechanisms and regulation are poorly understood). Once the choanoflagellate cell finds a suitable spot for settlement, all filopodia coalesce and sustain the secretion of an extracellular matrix that forms a cup-shaped lodge called a *theca* (Fig. 1D). Although this behavior has only been described in detail in two species so far, *Choanoeca perplexa* (Leadbeater 1977) and *S. rosetta* (Dayel et al. 2011), many choanoflagellates are capable of settlement and of theca secretion (Leadbeater 2014; Carr et al. 2017), which suggests this behavior might be more widespread across choanoflagellates.

The molecular pathways governing filopodial walking in choanoflagellates remain unknown. In animals, filopodial guidance notably involves voltage-gated calcium channels (Efremov et al. 2022). Intriguingly, axonal growth-cone motility generally relies on filopodial exploration of the environment (Wood and Martin 2002), and one might wonder whether axonal growth-cone guidance shares cellular mechanisms with choanoflagellate settlement.

Filopodial walking in choanoflagellates raises a number of open questions: how is locomotion accomplished? Does actin polymerization/depolymerization dynamics in filopodia underlie movement? Are the filopodia sensory? What stimuli, if any, inform the cellular decision to settle at a given spot? Theca secretion is a costly, complex and irreversible endeavor for the cell, and, once secreted, the theca can no longer be moved. What signaling pathway(s) implement the switch to irreversible settlement and theca secretion? We expect future investigations to shed light into some of these questions.

## Discussion: from microbial behavior to animal cognition

Although data remain scarce, it is already clear that choanoflagellates have complex perceptive abilities and behaviors: they display at least eight sensory modalities, and as many behavioral outputs (Fig. 2). Almost inevitably, many more must exist, as suggested by the complex molecular repertoire of putative sensory receptors encoded in the choanoflagellate genomes and transcriptomes.

Although the behavior of choanoflagellates is clearly complex, its complexity is probably similar to that of many other protists (Lyon et al. 2021), many of which have long been tractable behavioral models, for example, *Chlamydomonas* (Bennett and Golestanian 2015), *Dictyostelium* (King and Insall 2009)*, Physarum* (Nakagaki et al. 2000), *Paramecium* (Brette 2021) and other ciliates (e.g.,* Euplotes* (Larson et al. 2022) or *Lacrymaria* (Coyle et al. 2019)). It is thus legitimate to ask: are choanoflagellates especially relevant to understanding the origin and evolution of animal behavior? In our opinion, the answer is yes, because of the extensive set of molecular (and cellular) sensors and effectors they share with animal cells. For example, microvillar bundles are fundamental to animal perception and are only shared with choanoflagellates to the exclusion of other unicellular eukaryotes (Sebé-Pedrós et al. 2013; Peña et al. 2016; Brunet and King 2017). The lineage of unicellular ancestors that gave rise to animals might not initially have had a particularly extensive behavioral repertoire; but the evolutionary path that gave rise to animal cognition was inevitably constrained by the genetic background of the one single-celled lineage animals happen to have evolved from. If we want to understand the “evolutionary tinkering” (Jacob 1977) that culminated in the human or the octopus brains, we must thus start by understanding these humble origins.

What metazoan-specific innovations allowed the elaboration of animal cognition? A key step in the evolution toward multicellular complexity was the emergence of intercellular coordination. Since choanoflagellates display multicellular morphologies, they must negotiate the delicate transition between the behavior of a single cell and that of a colony. Counter-intuitively, this did not necessarily involve any cellular communication at first. Indeed, *S. rosetta* displays equally efficient chemotaxis as single cells or as rosette colonies (Kirkegaard et al. 2016a), and quantification of flagellar beating suggests neighboring flagella within rosettes do not synchronize or otherwise coordinate (Kirkegaard et al. 2016b). It has thus been suggested that *S. rosetta* behaves as an “aggregate random walker” (Kirkegaard et al. 2016b): all cells within a colony behave in the exact same way as they would if they were alone, but this is sufficient to maintain run-and-tumble chemotaxis at the multicellular scale. (Note, however, that cells within *S. rosetta* rosettes are linked by cytoplasmic bridges (Dayel et al. 2011) that might allow exchange of intracellular signals, suggesting intercellular communication might occur in other contexts.) Similarly, in both unicellular and multicellular volvocale algae, phototaxis relies on similar mechanisms at the cellular level (albeit with different adaptation kinetics), and the evolutionary transition to multicellularity thus does not seem to have required the “invention” of any intercellular communication mechanism to maintain phototaxis (Leptos et al. 2022). Finally, it has been argued that such aggregate behavior is even displayed by some metazoans, for example during ciliary locomotion by the placozoan *T. adhaerens*, where locomotory ciliated cells of the ventral surface were proposed to all perceive and react to chemoattractants independently (Smith et al. 2019). Other studies have, however, put forward evidence of coordination between placozoan cilia (Bull et al. 2021).

Although collective behaviors appear to be generated without intercellular communication, could some simple intercellular communication nonetheless exist in choanoflagellate colonies? No evidence for coordination has yet been obtained, but the question has been little studied and thus remains open. Potential contexts include inversion in the multicellular species *C. flexa*, where cell–cell communication might theoretically be ensured either by mechanical or chemical signals (the latter potentially including nitric oxide (Reyes-Rivera et al. 2022) or neuropeptides (Yañez-Guerra et al. 2022)). Another hallmark of many choanoflagellate colonies are cytoplasmic bridges, which might allow direct intercellular transfer of small molecules (such as calcium or cyclic nucleotides) (Dayel et al. 2011; Chaigne and Brunet 2022).

A deeper characterization of molecular and cellular sensors, processors and effectors in choanoflagellates might help inform and test “division-of-labor” scenarios for the origin of animal neuronal circuits (Mackie 1970; Arendt 2008). A recurrent theme might have been the conversion of environmental stimuli into intercellular signals. For example, many choanoflagellate genomes encode putative receptors for ATP and glutamate, which are presumably detected when they are generated by environmental sources, such as damaged neighboring cells. On the other hand, in animals, ATP and glutamate are actively secreted neurotransmitters. Thus, the evolution of intercellular communication might more generally have involved cells acquiring the ability to “mimic” external signals to stimulate their neighbors. Future comparative and functional research among more choanoflagellate and other unicellular species might inform how animals still function like “societies of microbes”, but also how metazoan-specific integrative mechanisms have given rise to the spectacular and unique behaviors of animals.

### Supplementary Information

Below is the link to the electronic supplementary material.

Supplementary file1 (DOCX 51 KB)

## Data Availability

Not applicable.

## References

[CR1] Alberts B (2008). Molecular biology of the cell.

[CR2] Alegado RA, Brown LW, Cao S (2012). A bacterial sulfonolipid triggers multicellular development in the closest living relatives of animals. Elife.

[CR3] Al-Sheikh U, Kang L (2020). Molecular crux of hair cell mechanotransduction machinery. Neuron.

[CR4] Andreakis N, D’Aniello S, Albalat R (2011). Evolution of the nitric oxide synthase family in metazoans. Mol Biol Evol.

[CR5] Arendt D (2003). Evolution of eyes and photoreceptor cell types. Int J Dev Biol.

[CR6] Arendt D (2008). The evolution of cell types in animals: emerging principles from molecular studies. Nat Rev Genet.

[CR7] Arendt D (2020). The Evolutionary assembly of neuronal machinery. Curr Biol.

[CR8] Armon S, Bull MS, Aranda-Diaz A, Prakash M (2018). Ultrafast epithelial contractions provide insights into contraction speed limits and tissue integrity. Proc Natl Acad Sci.

[CR9] Avelar GM, Schumacher RI, Zaini PA (2014). A Rhodopsin-guanylyl cyclase gene fusion functions in visual perception in a fungus. Curr Biol.

[CR10] Azmitia EC, Azmitia EC (2020). Evolution of serotonin: sunlight to suicide. Handbook of Behavioral Neuroscience.

[CR11] Bargmann CI (2006). Chemosensation in C elegans. WormBook.

[CR12] Bennett RR, Golestanian R (2015). A steering mechanism for phototaxis in *Chlamydomonas*. J R Soc Interface.

[CR13] Bezares-Calderón LA, Berger J, Jasek S (2018). Neural circuitry of a polycystin-mediated hydrodynamic startle response for predator avoidance. Elife.

[CR14] Bezares-Calderón LA, Berger J, Jékely G (2020). Diversity of cilia-based mechanosensory systems and their functions in marine animal behaviour. Phil Trans R Soc B.

[CR15] Block SM, Segall JE, Berg HC (1983). Adaptation kinetics in bacterial chemotaxis. J Bacteriol.

[CR16] Booth DS, King N (2020). Genome editing enables reverse genetics of multicellular development in the choanoflagellate Salpingoeca rosetta. Elife.

[CR17] Booth DS, Szmidt-Middleton H, King N (2018). Choanoflagellate transfection illuminates their cell biology and the ancestry of animal septins. Mol Biol.

[CR18] Brette R (2021). Integrative neuroscience of paramecium a swimming neuron. Neuro.

[CR19] Brown LS (2014). Eubacterial rhodopsins unique photosensors and diverse ion pumps. Biochimica Et Biophysica Acta (BBA)..

[CR20] Brunet T, Arendt D (2016). From damage response to action potentials: early evolution of neural and contractile modules in stem eukaryotes. Philos Trans R Soc Lond, b, Biol Sci.

[CR21] Brunet T, King N (2017). The origin of animal multicellularity and cell differentiation. Dev Cell.

[CR22] Brunet T, Larson BT, Linden TA (2019). Light-regulated collective contractility in a multicellular choanoflagellate. Science.

[CR23] Brunet T, Albert M, Roman W (2021). A flagellate-to-amoeboid switch in the closest living relatives of animals. Elife.

[CR24] Bull MS, Prakash VN, Prakash M (2021) Ciliary flocking and emergent instabilities enable collective agility in a non-neuromuscular anima. 10.48550/ARXIV.2107.02934

[CR25] Burkhardt P (2015). The origin and evolution of synaptic proteins - choanoflagellates lead the way. J Exp Biol.

[CR26] Burkhardt P, Stegmann CM, Cooper B (2011). Primordial neurosecretory apparatus identified in the choanoflagellate Monosiga brevicollis. Proc Natl Acad Sci USA.

[CR27] Burkhardt P, Grønborg M, McDonald K (2014). Evolutionary insights into premetazoan functions of the neuronal protein homer. Mol Biol Evol.

[CR28] Burki F, Roger AJ, Brown MW, Simpson AGB (2020). The new tree of eukaryotes. Trends Ecol Evol.

[CR29] Cai X (2008). Unicellular Ca2+ signaling ‘toolkit’ at the origin of metazoa. Mol Biol Evol.

[CR30] Cai X (2012). Evolutionary genomics reveals the premetazoan origin of opposite gating polarity in animal-type voltage-gated ion channels. Genomics.

[CR31] Cai X, Clapham DE (2012). Ancestral Ca2+ signaling machinery in early animal and fungal evolution. Mol Biol Evol.

[CR32] Carr M, Leadbeater BSC, Hassan R (2008). Molecular phylogeny of choanoflagellates, the sister group to Metazoa. Proc Natl Acad Sci USA.

[CR33] Carr M, Richter DJ, Fozouni P (2017). A six-gene phylogeny provides new insights into choanoflagellate evolution. Mol Phylogenet Evol.

[CR34] Carrasco-Pujante J, Bringas C, Malaina I (2021). Associative conditioning is a robust systemic behavior in unicellular organisms an interspecies comparison. Front Microbiol.

[CR35] Cavalier-Smith T (2017). Origin of animal multicellularity: precursors, causes, consequences-the choanoflagellate/sponge transition, neurogenesis and the Cambrian explosion. Philos Trans R Soc Lond B Biol Sci.

[CR36] Chaigne A, Brunet T (2022). Incomplete abscission and cytoplasmic bridges in the evolution of eukaryotic multicellularity. Curr Biol.

[CR37] Cheng K (2021). Learning in cnidaria: a systematic review. Learn Behav.

[CR38] Colasanti M, Persichini T, Venturini G (2010). Nitric oxide pathway in lower metazoans. Nitric Oxide.

[CR39] Colgren J, Burkhardt P (2022). The premetazoan ancestry of the synaptic toolkit and appearance of first neurons. Essays Biochem.

[CR40] Corey DP, Holt JR (2016). Are TMCs the mechanotransduction channels of vertebrate hair cells?. J Neurosci.

[CR41] Coyle SM, Flaum EM, Li H (2019). Coupled active systems encode an emergent hunting behavior in the unicellular predator lacrymaria olor. Curr Biol.

[CR42] Cutruzzolà F (1999). Bacterial nitric oxide synthesis. Biochem Biophys Acta.

[CR43] Dayel MJ, Alegado RA, Fairclough SR (2011). Cell differentiation and morphogenesis in the colony-forming choanoflagellate Salpingoeca rosetta. Dev Biol.

[CR44] de Mendoza A, Sebé-Pedrós A, Ruiz-Trillo I (2014). The Evolution of the GPCR signaling system in eukaryotes: modularity, conservation, and the transition to metazoan multicellularity. Genome Biol Evol.

[CR45] Dussutour A (2021). Learning in single cell organisms. Biochem Biophys Res Commun.

[CR46] Efremov AK, Yao M, Sun Y (2022). Application of piconewton forces to individual filopodia reveals mechanosensory role of L-type Ca2+ channels. Biomaterials.

[CR47] Erives A, Fritzsch B (2020). A screen for gene paralogies delineating evolutionary branching order of early Metazoa. Genes Genomes Genetics..

[CR48] Ernst OP, Lodowski DT, Elstner M (2014). Microbial and animal rhodopsins: structures, functions, and molecular mechanisms. Chem Rev.

[CR49] Fairclough SR, Chen Z, Kramer E (2013). Premetazoan genome evolution and the regulation of cell differentiation in the choanoflagellate Salpingoeca rosetta. Genome Biol.

[CR50] Fritzsch B, Straka H (2014). Evolution of vertebrate mechanosensory hair cells and inner ears: toward identifying stimuli that select mutation driven altered morphologies. J Comp Physiol A Neuroethol Sens Neural Behav Physiol.

[CR51] Fritzsch B, Beisel KW, Pauley S, Soukup G (2007). Molecular evolution of the vertebrate mechanosensory cell and ear. Int J Dev Biol.

[CR52] Fujiu K, Nakayama Y, Yanagisawa A (2009). Chlamydomonas CAV2 encodes a voltage- dependent calcium channel required for the flagellar waveform conversion. Curr Biol.

[CR53] Fujiu K, Nakayama Y, Iida H (2011). Mechanoreception in motile flagella of Chlamydomonas. Nat Cell Biol.

[CR54] Galindo LJ, Milner DS, Gomes SL, Richards TA (2022). A light-sensing system in the common ancestor of the fungi. Curr Biol.

[CR55] Geffeney SL, Goodman MB (2012). How We Feel: Ion channel partnerships that detect mechanical inputs and give rise to touch and pain perception. Neuron.

[CR56] Gershman SJ, Balbi PE, Gallistel CR, Gunawardena J (2021). Reconsidering the evidence for learning in single cells. Elife.

[CR57] Grau-Bové X, Torruella G, Donachie S (2017). Dynamics of genomic innovation in the unicellular ancestry of animals. Elife.

[CR58] Grote M, Engelhard M, Hegemann P (2014). Of ion pumps, sensors and channels perspectives on microbial rhodopsins between science and history. Biochem Biophys Acta.

[CR59] Hake KH, West PT, McDonald K (2021). Colonial choanoflagellate isolated from Mono Lake harbors a microbiome. Biophysica Acta.

[CR60] Hardie RC, Franze K (2012). Photomechanical Responses in *Drosophila* Photoreceptors. Science.

[CR61] Harz H, Hegemann P (1991). Rhodopsin-regulated calcium currents in Chlamydomonas. Nature.

[CR62] He L, Si G, Huang J (2018). Mechanical regulation of stem-cell differentiation by the stretch-activated Piezo channel. Nature.

[CR63] Hehenberger E, Tikhonenkov DV, Kolisko M (2017). Novel predators reshape holozoan phylogeny and reveal the presence of a two-component signaling system in the ancestor of animals. Curr Biol.

[CR64] Himmel NJ, Cox DN (2020). Transient receptor potential channels: current perspectives on evolution, structure, function and nomenclature. Royal Soci Biol Sci.

[CR65] Höfer D, Drenckhahn D (1999). Localisation of actin, villin, fimbrin, ezrin and ankyrin in rat taste receptor cells. Histochemistry.

[CR66] Hofmann KP, Scheerer P, Hildebrand PW (2009). A G protein-coupled receptor at work: the rhodopsin model. Trends Biochem Sci.

[CR67] Holland EM, Harz H, Uhl R, Hegemann P (1997). Control of phobic behavioral responses by rhodopsin-induced photocurrents in Chlamydomonas. Biophys J.

[CR68] Inaba K (2015). Calcium sensors of ciliary outer arm dynein: functions and phylogenetic considerations for eukaryotic evolution. Cilia.

[CR69] Inglis PN, Ou G, Leroux MR, Scholey JM (2018). The sensory cilia of Caenorhabditis elegans. WormBook..

[CR70] Inoue K, Kato Y, Kandori H (2015). Light-driven ion-translocating rhodopsins in marine bacteria. Trends Microbiol.

[CR71] Jacob F (1977). Evolution and tinkering. Science.

[CR72] James-Clark H (1867). Conclusive proofs of the animality of the ciliate sponges, and of their affinities with the Infusoria flagellata. J Nat Hist.

[CR73] Jeans JH (1968). Science and music.

[CR74] Jékely G (2019). Evolution: how not to become an animal. Curr Biol.

[CR75] Jenkins PM, McEwen DP, Martens JR (2009). Olfactory cilia: linking sensory cilia function and human disease. Chem Senses.

[CR76] Jia Y, Zhao Y, Kusakizako T (2020). TMC1 and TMC2 proteins are pore-forming subunits of mechanosensitive ion channels. Neuron.

[CR77] Jikeli JF, Alvarez L, Friedrich BM (2015). Sperm navigation along helical paths in 3D chemoattractant landscapes. Nat Commun.

[CR78] Johnson J-LF, Leroux MR (2010). cAMP and cGMP signaling: sensory systems with prokaryotic roots adopted by eukaryotic cilia. Trends Cell Biol.

[CR79] Jung K-H, Trivedi VD, Spudich JL (2003). Demonstration of a sensory rhodopsin in eubacteria: Sensory rhodopsin in eubacteria. Mol Microbiol.

[CR80] King N, Carroll SB (2001). A receptor tyrosine kinase from choanoflagellates: molecular insights into early animal evolution. Proc Natl Acad Sci USA.

[CR81] King JS, Insall RH (2009). Chemotaxis: finding the way forward with dictyostelium. Trends Cell Biol.

[CR82] King N, Westbrook MJ, Young SL (2008). The genome of the choanoflagellate Monosiga brevicollis and the origin of metazoans. Nature.

[CR83] Kirkegaard JB, Bouillant A, Marron AO (2016). Aerotaxis in the closest relatives of animals. Elife.

[CR84] Kirkegaard JB, Marron AO, Goldstein RE (2016). Motility of Colonial Choanoflagellates and the Statistics of Aggregate Random Walkers. Phys Rev Lett.

[CR85] Knoll AH (2011). The multiple origins of complex multicellularity. Annu Rev Earth Planet Sci.

[CR86] Koehl M (2021). Selective factors in the evolution of multicellularity in choanoflagellates. J Exp Zool B Mol Dev Evol.

[CR87] Koyanagi M, Terakita A (2014). Diversity of animal opsin-based pigments and their optogenetic potential. Biochem Biophys Acta.

[CR88] Kumler WE, Jorge J, Kim PM (2020). Does formation of multicellular colonies by choanoflagellates affect their susceptibility to capture by passive protozoan predators?. J Eukaryot Microbiol.

[CR89] Larson BT, Garbus J, Pollack JB, Marshall WF (2022). A unicellular walker controlled by a microtubule-based finite-state machine. Curr Biol.

[CR90] Laundon D, Larson BT, McDonald K (2019). The architecture of cell differentiation in choanoflagellates and sponge choanocytes. PLoS Biol.

[CR91] Leadbeater BSC (1977). Observations on the life-history and ultrastructure of the marine choanoflagellate *Choanoeca perplexa* Ellis. J Mar Biol Ass.

[CR92] Leadbeater BS (1983). Life-history and ultrastructure of a new marine species of Proterospongia (Choanoflagellida). J Mar Biol Assoc UK.

[CR93] Leadbeater BSC (1983). Distribution and chemistry of microfilaments in choanoflagellates, with special reference to the collar and other tentacle systems. Protistologica.

[CR94] Leadbeater BSC (2014). The Choanoflagellates: Evolution.

[CR95] Leptos KC, Chioccioli M, Furlan S (2022). Phototaxis of Chlamydomonas arises from a tuned adaptive photoresponse shared with multicellular Volvocine green algae. Phys Rev E.

[CR96] Levayer R, Lecuit T (2012). Biomechanical regulation of contractility: spatial control and dynamics. Trends Cell Biol.

[CR97] Levin TC, King N (2013). Evidence for sex and recombination in the choanoflagellate Salpingoeca rosetta. Curr Biol.

[CR98] Liebeskind BJ, Hillis DM, Zakon HH (2011). Evolution of sodium channels predates the origin of nervous systems in animals. Proc Natl Acad Sci.

[CR99] Loy I, Carnero-Sierra S, Acebes F (2021). Where association ends. A review of associative learning in invertebrates, plants and protista, and a reflection on its limits. J Exp Psychol Anim Learn Cogn.

[CR100] Lyon P, Keijzer F, Arendt D, Levin M (2021). Reframing cognition: getting down to biological basics. Philosoph Trans Royal Soc.

[CR101] Mackie GO (1970). Neuroid conduction and the evolution of conducting tissues. Q Rev Biol.

[CR102] Mackin KA, Roy RA, Theobald DL (2014). An empirical test of convergent evolution in rhodopsins. Mol Biol Evol.

[CR103] Mah JL, Leys SP (2017). Think like a sponge: The genetic signal of sensory cells in sponges. Dev Biol.

[CR104] Manning G, Young SL, Miller WT, Zhai Y (2008). The protist, Monosiga brevicollis, has a tyrosine kinase signaling network more elaborate and diverse than found in any known metazoan. Proc Natl Acad Sci.

[CR105] Martens-Habbena W, Qin W, Horak REA (2015). The production of nitric oxide by marine ammonia-oxidizing archaea and inhibition of archaeal ammonia oxidation by a nitric oxide scavenger: Marine AOA produce NO and are inhibited by NO scavenger. Environ Microbiol.

[CR106] Martin AC, Goldstein B (2014). Apical constriction: themes and variations on a cellular mechanism driving morphogenesis. Development.

[CR107] Mattila PK, Lappalainen P (2008). Filopodia: molecular architecture and cellular functions. Nat Rev Mol Cell Biol.

[CR108] Miller WT (2012). Tyrosine kinase signaling and the emergence of multicellularity. Biochem Biophys Acta.

[CR109] Mills DB, Francis WR, Vargas S (2018). The last common ancestor of animals lacked the HIF pathway and respired in low-oxygen environments. Elife.

[CR110] Miño GL, Koehl M (2017). Finding patches in a heterogeneous aquatic environment: pH-taxis by the dispersal stage of choanoflagellates. Limnol Oceanog Lett.

[CR111] Mitchell DR (2007). The Evolution of Eukaryotic Cilia and Flagella as Motile and Sensory Organelles. Eukaryotic Membranes and Cytoskeleton.

[CR112] Moran Y, Barzilai MG, Liebeskind BJ, Zakon HH (2015). Evolution of voltage-gated ion channels at the emergence of Metazoa. J Exp Biol.

[CR113] Moroz LL, Romanova DY, Nikitin MA (2020). The diversification and lineage-specific expansion of nitric oxide signaling in Placozoa: insights in the evolution of gaseous transmission. Sci Rep.

[CR114] Moroz LL, Romanova DY, Kohn AB (2021). Neural versus alternative integrative systems: molecular insights into origins of neurotransmitters. Phil Trans R Soc B.

[CR115] Murthy SE, Dubin AE, Patapoutian A (2017). Piezos thrive under pressure: mechanically activated ion channels in health and disease. Nat Rev Mol Cell Biol Adv.

[CR116] Nakagaki T, Yamada H, Tóth Á (2000). Maze-solving by an amoeboid organism. Nature.

[CR117] Needham DM, Yoshizawa S, Hosaka T (2019). A distinct lineage of giant viruses brings a rhodopsin photosystem to unicellular marine predators. Proc Natl Acad Sci.

[CR118] Needham DM, Poirier C, Bachy C (2022). The microbiome of a bacterivorous marine choanoflagellate contains a resource-demanding obligate bacterial associate. Nat Microbiol.

[CR119] Nielsen C (2012). Animal Evolution: Interrelationships of the Living Phyla.

[CR120] Nilsson D-E (2009). The evolution of eyes and visually guided behaviour. Phil Trans R Soc B.

[CR121] Palczewski K (2006). G Protein-coupled receptor rhodopsin. Annu Rev Biochem.

[CR122] Pan B, Akyuz N, Liu X-P (2018). TMC1 forms the pore of mechanosensory transduction channels in vertebrate inner ear hair cells. Neuron.

[CR123] Peña JF, Alié A, Richter DJ (2016). Conserved expression of vertebrate microvillar gene homologs in choanocytes of freshwater sponges. EvoDevo.

[CR124] Peng G, Shi X, Kadowaki T (2015). Evolution of TRP channels inferred by their classification in diverse animal species. Mol Phylogenet Evol.

[CR125] Prieto-Echagüe V, Chan PM, Craddock BP (2011). PTB domain-directed substrate targeting in a tyrosine kinase from the unicellular Choanoflagellate Monosiga brevicollis. PLoS ONE.

[CR126] Ranade SS, Woo S-H, Dubin AE (2014). Piezo2 is the major transducer of mechanical forces for touch sensation in mice. Nature.

[CR127] Reyes-Rivera J, Wu Y, Guthrie BGH (2022). Nitric oxide signaling controls collective contractions in a colonial choanoflagellate. Curr Biol.

[CR128] Rosa N, Shabardina V, Ivanova H (2021). Tracing the evolutionary history of Ca2+-signaling modulation by human Bcl-2: Insights from the Capsaspora owczarzaki IP3 receptor ortholog. Biochem Biophys Acta.

[CR129] Ros-Rocher N, Pérez-Posada A, Leger MM, Ruiz-Trillo I (2021). The origin of animals: an ancestral reconstruction of the unicellular-to-multicellular transition. Open Biol.

[CR130] Ruiz-Trillo I, Roger AJ, Burger G (2008). A phylogenomic investigation into the origin of metazoa. Mol Biol Evol.

[CR131] Rutaganira FUN, Scopton AP, Dar AC, King N (2022). A small molecule screen reveals the essentiality of kinase for choanoflagellate. Cell Prolif.

[CR132] Saville-Kent W (1880). A manual of the Infusoria: including a description of all known Flagellate, Ciliate, and Tentaculiferous Protozoa, British and foreign, and an account of the organization and the affinities of the sponges. D Bogue..

[CR133] Sebé-Pedrós A, Burkhardt P, Sánchez-Pons N (2013). Insights into the origin of metazoan filopodia and microvilli. Mol Biol Evol.

[CR134] Sebé-Pedrós A, Degnan BM, Ruiz-Trillo I (2017). The origin of Metazoa: a unicellular perspective. Nat Rev Genet.

[CR135] Shichida Y, Morizumi T (2006). Mechanism of G-protein Activation by Rhodopsin. Photochem Photobiol.

[CR136] Sigg MA, Menchen T, Lee C (2017). Evolutionary proteomics uncovers ancient associations of cilia with signaling pathways. Dev Cell.

[CR137] Smith DV, Margolskee RF (2001). Making sense of taste. Sci Am.

[CR138] Smith CL, Reese TS, Govezensky T, Barrio RA (2019). Coherent directed movement toward food modeled in Trichoplax, a ciliated animal lacking a nervous system. Proc Natl Acad Sci USA.

[CR139] Stocker R (2012). Marine microbes see a sea of gradients. Science.

[CR140] Stocker R, Seymour JR, Samadani A (2008). Rapid chemotactic response enables marine bacteria to exploit ephemeral microscale nutrient patches. Proc Natl Acad Sci.

[CR141] Suga H, Sasaki G, Kuma K (2008). Ancient divergence of animal protein tyrosine kinase genes demonstrated by a gene family tree including choanoflagellate genes. FEBS Lett.

[CR142] Suga H, Dacre M, de Mendoza A (2012). Genomic Survey of premetazoans shows deep conservation of cytoplasmic tyrosine kinases and multiple radiations of receptor tyrosine kinases. Sci Sig..

[CR143] Tanaka K, Choi J, Cao Y, Stacey G (2014). Extracellular ATP acts as a damage-associated molecular pattern (DAMP) signal in plants. Front Plant Sci.

[CR144] Tian Y, Gao S, Yang S, Nagel G (2018). A novel rhodopsin phosphodiesterase from Salpingoeca rosetta shows light-enhanced substrate affinity. Biochemical Journal.

[CR145] Torruella G, de Mendoza A, Grau-Bové X (2015). Phylogenomics reveals convergent evolution of lifestyles in close relatives of animals and fungi. Curr Biol.

[CR146] Trautmann A (2009). Extracellular ATP in the immune system: more than just a “danger signal”. Sci Sig..

[CR147] Vidal B, Aghayeva U, Sun H (2018). An atlas of Caenorhabditis elegans chemoreceptor expression. PLoS Biol.

[CR148] von Uexküll J (1934). A foray into the worlds of animals and humans: with A theory of meaning, 1st University of Minnesota.

[CR149] Walsh CM, Bautista DM, Lumpkin EA (2015). Mammalian touch catches up. Curr Opin Neurobiol.

[CR150] Wetzel LA, Levin TC, Hulett RE (2018). Predicted glycosyltransferases promote development and prevent spurious cell clumping in the choanoflagellate S. Rosetta. Elife.

[CR151] Wood W, Martin P (2002). Structures in focus—filopodia. Int J Biochem Cell Biol.

[CR152] Woznica A, Cantley AM, Beemelmanns C (2016). Bacterial lipids activate, synergize, and inhibit a developmental switch in choanoflagellates. Proc Natl Acad Sci USA.

[CR154] Woznica A, Gerdt JP, Hulett RE (2017). Mating in the Closest Living Relatives of Animals Is Induced by a Bacterial Chondroitinase. Cell.

[CR155] Woznica A, Kumar A, Sturge CR (2021). STING mediates immune responses in the closest living relatives of animals. Elife.

[CR157] Yañez-Guerra LA, Thiel D, Jékely G (2022). Premetazoan origin of neuropeptide signaling. Mol Biol Evol.

[CR158] Yong E (2022). An Immense World: How Animal Senses Reveal the Hidden Realms Around Us. Peng Rand House..

[CR159] Yoshida K, Tsunoda SP, Brown LS, Kandori H (2017). A unique choanoflagellate enzyme rhodopsin exhibits light-dependent cyclic nucleotide phosphodiesterase activity. J Biol Chem.

