# Supplementary material for: What is it like to be a choanoflagellate? Sensation, processing and behavior in the closest unicellular relatives of animals
Source: Anim Cogn. 2023 Apr 17;26(6):1767–82. doi: 10.1007/s10071-023-01776-z (PMC10770216; doi:10.1007/s10071-023-01776-z)
Supplement: Supplementary file 1 — Supplementary file1 (DOCX 51 KB) [file 10071_2023_1776_MOESM1_ESM.docx]

**Table S1. Molecular sensors and sensory transducers in choanozoans (related to Fig. 3).** Shown are reported ortholog sequences in literature or accession numbers of orthologs predicted here. Putative orthologs were identified as reciprocal best BlastP hits and with PfamScan using the gathering threshold as cutoff value (Mistry et al. 2021). Most BLAST searches were conducted against the nonredundant protein database (nr) of the National Center for Biotechnology Information (NCBI). The *C. flexa* predicted proteome was searched locally (using Blast version 2.3.0) using *S. rosetta* sequences as query.

| **Sensory molecules** | **Orthologs in *M. brevicollis*** | **Orthologs in *C. flexa*** | **Orthologs in *S. rosetta*** |
| --- | --- | --- | --- |
| Phoenixin (PNX) | *Absent from genome* (Yañez-Guerra et al. 2022) | Accession number in transcriptome: TRINITY_DN1992_c0_g1_i1 (absent from the predicted proteome due to short length) | (Yañez-Guerra et al. 2022) |
| Nesfatin | (Yañez-Guerra et al. 2022) | *Absent from transcriptome* | (Yañez-Guerra et al. 2022) |
| Hypoxia-inducible factor (HIF) | *Absent from genome* (Mills et al. 2018) | *Absent from transcriptome* | *Absent from genome* (Mills et al. 2018) |
| Eukaryotic NO synthase | *Absent from genome* (Reyes-Rivera et al. 2022) | *Absent from transcriptome* (Reyes-Rivera et al. 2022) | *Absent from genome* (Reyes-Rivera et al. 2022) |
| Prokaryotic NO synthase | *Absent from genome* (Reyes-Rivera et al. 2022) | (Reyes-Rivera et al. 2022) | *Absent from genome* (Reyes-Rivera et al. 2022) |
| NO receptor (soluble guanylyl cyclase) | Accession numbers: MONBRDRAFT_8342, MONBRDRAFT_31218, MONBRDRAFT_32325, MONBRDRAFT_34163, MONBRDRAFT_38443, MONBRDRAFT_34366, MONBRDRAFT_29467, MONBRDRAFT_9289, MONBRDRAFT_26544, MONBRDRAFT_37593, MONBRDRAFT_37592, MONBRDRAFT_32949, MONBRDRAFT_34682, MONBRDRAFT_27986, MONBRDRAFT_33782, MONBRDRAFT_37136, MONBRDRAFT_14998, MONBRDRAFT_14699 | (Reyes-Rivera et al. 2022) | (Reyes-Rivera et al. 2022) |
| Ionotropic ATP receptor (P2X) | (Fountain et al. 2008; Fountain and Burnstock 2009; Cai 2012a) | Accession numbers in proteome: TRINITY_DN11075_c0_g1_i4.p2, TRINITY_DN10750_c0_g1_i7.p1 | (Fountain et al. 2008; Fountain and Burnstock 2009; Cai 2012a) |
| Ionotropic glutamate/ glycine/serotonin receptor | *Absent from genome* | (Brunet et al. 2019) | *Absent from genome* |
| Tyrosine kinases | (King et al. 2008; Manning et al. 2008) | *>90 predicted in transcriptome* | (King et al. 2008; Manning et al. 2008) |
| Metazoan-type rhodopsins | *Absent from genome* (Feuda et al. 2012) | *Absent from transcriptome* | *Absent from genome* (Feuda et al. 2012) |
| Microbial-type rhodopsins | *Absent from genome* | (Brunet et al. 2019) | (Spudich 2006; Avelar et al. 2014; Brunet et al. 2019; Galindo et al. 2022) |
| Piezo | (Fairclough et al. 2013; Prole and Taylor 2013) | accession number in proteome: TRINITY_DN9110_c0_g1_i1.p1 | (Fairclough et al. 2013; Prole and Taylor 2013) |
| DEG/ENaC | *Absent from genome* | (Brunet et al. 2019) | *Absent from genome* |
| TMC | (Erives and Fritzsch 2020) | accession numbers in proteome: TRINITY_DN10991_c0_g2_i1.p1, TRINITY_DN6580_c0_g1_i2.p1 | (Erives and Fritzsch 2020) |
| GPCRs | (de Mendoza et al. 2014) | accession numbers in proteome: TRINITY_DN11241_c1_g2_i2.p1, TRINITY_DN11246_c0_g1_i3.p1, TRINITY_DN18375_c0_g1_i1.p1, TRINITY_DN11989_c0_g1_i23.p1, TRINITY_DN11695_c0_g1_i3.p1, TRINITY_DN10894_c0_g1_i1.p1 | (de Mendoza et al. 2014) |
| Heterotrimeric G proteins | (de Mendoza et al. 2014) | accession number in proteome: TRINITY_DN7384_c0_g1_i1.p1, TRINITY_DN7759_c0_g1_i1.p1, TRINITY_DN9679_c0_g2_i7.p1, TRINITY_DN12400_c0_g1_i3.p1 | (de Mendoza et al. 2014) |
| Trp channels | (Cai 2008; Peng et al. 2015; Himmel and Cox 2020) | accession numbers in proteome: TRINITY_DN7699_c0_g1_i3.p1, TRINITY_DN12084_c3_g3_i1.p1 | (Cai 2008; Peng et al. 2015; Himmel and Cox 2020) |
| Potassium voltage-gated (KV) channels | (Moran et al. 2015) | accession numbers in proteome: TRINITY_DN7967_c0_g2_i2.p2, TRINITY_DN9681_c0_g1_i2.p1, TRINITY_DN11869_c4_g1_i1.p1 | *Absent from genome* |
| Calcium voltage-gated (CaV) channels | (Liebeskind et al. 2011; Moran et al. 2015) | accession numbers in proteome: TRINITY_DN11215_c0_g1_i3.p1, TRINITY_DN11905_c0_g1_i8.p1 | (Liebeskind et al. 2011; Moran et al. 2015) |
| Sodium voltage-gated (NaV) channels | (Liebeskind et al. 2011; Moran et al. 2015) | accession number in proteome: TRINITY_DN11905_c0_g1_i8.p1 | *Absent from genome* |
| Cyclic nucleotide-gated (CNG) channels | (Cai 2012b; Reyes-Rivera et al. 2022) | (Cai 2012b; Reyes-Rivera et al. 2022) | (Cai 2012b; Reyes-Rivera et al. 2022) |
| Phosphodiesterase | accession number: MONBRDRAFT_9955 | (Brunet et al. 2019) | (Fairclough et al. 2013) |
| Phospholipase C (PLC) | See NCBI database | accession numbers in proteome: TRINITY_DN10872_c0_g1_i3.p1, TRINITY_DN10890_c0_g1_i2.p1, TRINITY_DN11963_c0_g1_i1.p1, TRINITY_DN7664_c1_g1_i1.p1, TRINITY_DN5227_c1_g1_i1.p1, TRINITY_DN8394_c0_g2_i2.p1, TRINITY_DN9983_c0_g1_i1.p1, TRINITY_DN9728_c0_g2_i2.p1, TRINITY_DN8399_c0_g1_i1.p1 | (Fairclough et al. 2013) |
| IP3 receptor | (Cai and Clapham 2012; Burkhardt et al. 2014; Rosa et al. 2021) | accession numbers in proteome: TRINITY_DN11373_c0_g1_i2.p1, TRINITY_DN11373_c0_g1_i2.p1, TRINITY_DN11178_c0_g1_i4.p1, TRINITY_DN10657_c0_g1_i3.p1 | (Cai and Clapham 2012; Burkhardt et al. 2014; Rosa et al. 2021) |

**References for Table S1**

Avelar GM, Schumacher RI, Zaini PA, et al (2014) A Rhodopsin-Guanylyl Cyclase Gene Fusion Functions in Visual Perception in a Fungus. Current Biology 24:1234–1240. https://doi.org/10.1016/j.cub.2014.04.009

Brunet T, Larson BT, Linden TA, et al (2019) Light-regulated collective contractility in a multicellular choanoflagellate. Science 366:326–334. https://doi.org/10.1126/science.aay2346

Burkhardt P, Grønborg M, McDonald K, et al (2014) Evolutionary insights into premetazoan functions of the neuronal protein homer. Mol Biol Evol 31:2342–2355. https://doi.org/10.1093/molbev/msu178

Cai X (2012a) P2X receptor homologs in basal fungi. Purinergic Signal 8:11–13. https://doi.org/10.1007/s11302-011-9261-8

Cai X (2008) Unicellular Ca2+ Signaling ‘Toolkit’ at the Origin of Metazoa. Molecular Biology and Evolution 25:1357–1361. https://doi.org/10.1093/molbev/msn077

Cai X (2012b) Evolutionary genomics reveals the premetazoan origin of opposite gating polarity in animal-type voltage-gated ion channels. Genomics 99:241–245. https://doi.org/10.1016/j.ygeno.2012.01.007

Cai X, Clapham DE (2012) Ancestral Ca2+ Signaling Machinery in Early Animal and Fungal Evolution. Molecular Biology and Evolution 29:91–100. https://doi.org/10.1093/molbev/msr149

de Mendoza A, Sebé-Pedrós A, Ruiz-Trillo I (2014) The Evolution of the GPCR Signaling System in Eukaryotes: Modularity, Conservation, and the Transition to Metazoan Multicellularity. Genome Biology and Evolution 6:606–619. https://doi.org/10.1093/gbe/evu038

Erives A, Fritzsch B (2020) A Screen for Gene Paralogies Delineating Evolutionary Branching Order of Early Metazoa. G3 Genes|Genomes|Genetics 10:811–826. https://doi.org/10.1534/g3.119.400951

Fairclough SR, Chen Z, Kramer E, et al (2013) Premetazoan genome evolution and the regulation of cell differentiation in the choanoflagellate Salpingoeca rosetta. Genome Biol 14:R15. https://doi.org/10.1186/gb-2013-14-2-r15

Feuda R, Hamilton SC, McInerney JO, Pisani D (2012) Metazoan opsin evolution reveals a simple route to animal vision. Proc Natl Acad Sci USA 109:18868–18872. https://doi.org/10.1073/pnas.1204609109

Fountain SJ, Burnstock G (2009) An evolutionary history of P2X receptors. Purinergic Signal 5:269–272. https://doi.org/10.1007/s11302-008-9127-x

Fountain SJ, Cao L, Young MT, North RA (2008) Permeation Properties of a P2X Receptor in the Green Algae Ostreococcus tauri*. Journal of Biological Chemistry 283:15122–15126. https://doi.org/10.1074/jbc.M801512200

Galindo LJ, Milner DS, Gomes SL, Richards TA (2022) A light-sensing system in the common ancestor of the fungi. Current Biology 32:3146-3153.e3. https://doi.org/10.1016/j.cub.2022.05.034

Himmel NJ, Cox DN (2020) Transient receptor potential channels: current perspectives on evolution, structure, function and nomenclature. Proceedings of the Royal Society B: Biological Sciences 287:20201309. https://doi.org/10.1098/rspb.2020.1309

King N, Westbrook MJ, Young SL, et al (2008) The genome of the choanoflagellate Monosiga brevicollis and the origin of metazoans. Nature 451:783–788. https://doi.org/10.1038/nature06617

Liebeskind BJ, Hillis DM, Zakon HH (2011) Evolution of sodium channels predates the origin of nervous systems in animals. Proceedings of the National Academy of Sciences 108:9154–9159. https://doi.org/10.1073/pnas.1106363108

Manning G, Young SL, Miller WT, Zhai Y (2008) The protist, Monosiga brevicollis, has a tyrosine kinase signaling network more elaborate and diverse than found in any known metazoan. Proceedings of the National Academy of Sciences 105:9674–9679. https://doi.org/10.1073/pnas.0801314105

Mills DB, Francis WR, Vargas S, et al (2018) The last common ancestor of animals lacked the HIF pathway and respired in low-oxygen environments. eLife 7:e31176. https://doi.org/10.7554/eLife.31176

Mistry J, Chuguransky S, Williams L, et al (2021) Pfam: The protein families database in 2021. Nucleic Acids Research 49:D412–D419. https://doi.org/10.1093/nar/gkaa913

Moran Y, Barzilai MG, Liebeskind BJ, Zakon HH (2015) Evolution of voltage-gated ion channels at the emergence of Metazoa. Journal of Experimental Biology 218:515–525. https://doi.org/10.1242/jeb.110270

Peng G, Shi X, Kadowaki T (2015) Evolution of TRP channels inferred by their classification in diverse animal species. Molecular Phylogenetics and Evolution 84:145–157. https://doi.org/10.1016/j.ympev.2014.06.016

Prole DL, Taylor CW (2013) Identification and Analysis of Putative Homologues of Mechanosensitive Channels in Pathogenic Protozoa. PLoS One 8:e66068. https://doi.org/10.1371/journal.pone.0066068

Reyes-Rivera J, Wu Y, Guthrie BGH, et al (2022) Nitric oxide signaling controls collective contractions in a colonial choanoflagellate. Current Biology 32:2539-2547.e5. https://doi.org/10.1016/j.cub.2022.04.017

Rosa N, Shabardina V, Ivanova H, et al (2021) Tracing the evolutionary history of Ca2+-signaling modulation by human Bcl-2: Insights from the Capsaspora owczarzaki IP3 receptor ortholog. Biochimica et Biophysica Acta (BBA) - Molecular Cell Research 1868:119121. https://doi.org/10.1016/j.bbamcr.2021.119121

Spudich JL (2006) The multitalented microbial sensory rhodopsins. Trends in Microbiology 14:480–487. https://doi.org/10.1016/j.tim.2006.09.005

Yañez-Guerra LA, Thiel D, Jékely G (2022) Premetazoan Origin of Neuropeptide Signaling. Molecular Biology and Evolution 39:msac051. https://doi.org/10.1093/molbev/msac051
